# Antihyperlipidemic effects of *Pleurotus ostreatus *(oyster mushrooms) in HIV-infected individuals taking antiretroviral therapy

**DOI:** 10.1186/1472-6882-11-60

**Published:** 2011-08-10

**Authors:** Donald I Abrams, Paul Couey, Starley B Shade, Mary Ellen Kelly, Nnemdi Kamanu-Elias, Paul Stamets

**Affiliations:** 1 Hematology-Oncology, University of California San Francisco, Ward 84, 995 Potrero, San Francisco, CA 94110, USA; 2 Hematology-Oncology, University of California San Francisco, Ward 82, 995 Potrero, San Francisco, CA 94110, USA; 3 Center for AIDS Prevention Studies, University of California, San Francisco, 50 Beale Street, Suite 1300, San Francisco, CA 94105, USA; 4 Hematology-Oncology, University of California San Francisco, Ward 82, 995 Potrero, San Francisco, CA 94110, USA; 5 Positive Health Program, University of California San Francisco, Ward 84, 995 Potrero, San Francisco, CA 94110, USA; 6 Fungi Perfecti, LLC, Kamilche Point, WA 98507, USA

## Abstract

**Background:**

Antiretroviral treatment (ART) regimens in HIV patients commonly cause significant lipid elevations, including increases in both triglycerides and cholesterol. Standard treatments for hypercholesterolemia include the HMG CoA reductase inhibitors, or "statins." Because many ART agents and statins share a common metabolic pathway that uses the cytochrome P450 enzyme system, coadministration of ART with statins could increase statin plasma levels significantly. The oyster mushroom, *Pleurotus ostreatus*, has been shown in animal models to decrease lipid levels - a finding that has been supported by preliminary data in a small human trial.

**Methods:**

To assess the safety and efficacy of *P. ostreatus *in patients with HIV and ART-induced hyperlipidemia, a single-arm, open-label, proof-of-concept study of 8 weeks' duration with a target enrollment of 20 subjects was conducted. Study patients with ART-induced elevated non-HDL cholesterol levels (> 160 mg/dL) were enrolled. Participants received packets of freeze-dried *P. ostreatus *(15 gm/day) to be administered orally for the 8 week trial period. Lipid levels were drawn every two weeks to assess efficacy. Safety assessments included self-reported incidence of muscle aches and measurement of liver and muscle enzymes. Mean within-person change in lipid levels were estimated using generalized estimating equations to account for repeated observations on individuals. A 30 mg/dL decrease in non-HDL cholesterol was deemed clinically significant.

**Results:**

126 patients were screened to enroll 25, of which 20 completed the 8-week study. The mean age was 46.4 years (36-60). Patients had a mean 13.7 yrs of HIV infection. Mean non-HDL cholesterol was 204.5 mg/dL at day 0 and 200.2 mg/dL at day 56 (mean within-person change = -1.70; 95% confidence interval (CI) = -17.4, 14.0). HDL cholesterol levels increased from 37.8 mg/dL at day 0 to 40.4 mg/dL on day 56 (mean within-person change = 2.6; 95% CI = -0.1, 5.2). Triglycerides dropped from 336.4 mg/dL on day 0 to 273.4 mg/dL on day 56 (mean within-person change = -63.0; 95% CI = -120.9, -5.1). Only 3 individuals achieved a sustained clinically significant (30 mg/dL) decline in non-HDL cholesterol after 8 weeks of therapy. There were no adverse experiences reported other than patients' distaste for the preparation. Liver function tests and muscle enzymes were not affected by the 8 weeks of treatment.

**Conclusions:**

*Pleurotus ostreatus *as administered in this experiment did not lower non-HDL cholesterol in HIV patients with ART-induced hypercholesterolemia. Small changes in HDL and triglycerides were not of a clinical magnitude to warrant further study.

**Trial Registration:**

clinicaltrials.gov Identifier: NCT00069524

## Background

Combination antiretroviral therapy (ART) that includes a protease inhibitor (PI) or non-nucleoside reverse transcriptase inhibitor (NNRTI) is immensely effective in reducing plasma human immunodeficiency virus type 1(HIV) levels in most patients, thereby dramatically decreasing HIV disease progression and mortality. Unfortunately, ART is associated with a number of complex metabolic disturbances, including dyslipidemia, insulin resistance, and body fat redistribution [1,2]. The dyslipidemia is typically manifested as decreased HDL cholesterol, increased LDL cholesterol (including small, dense LDL), and increased total cholesterol. Such values, associated with increased cardiovascular risk in persons who are not HIV-infected, also place HIV patients at risk for premature cardiovascular events [3].

If dietary changes and exercise do not successfully reverse or arrest lipid changes, the clinician may consider altering the ART regimen; however, both PI and NNRTI-based therapies have been implicated in ART-related cholesterol elevations [4]. Even at the low 100 mg dose widely used to "boost" serum concentrations of other PIs, the PI ritonavir is known to significantly raise total and LDL cholesterol levels [5]. Hyperlipidemia is most commonly treated with 3-hydroxy-3methylglutaryl coenzyme A (HMG CoA) reductase inhibitors, or "statins." Using statins concomitantly with ART, though, is complicated by the fact that statin metabolism is dependant on the same cytochrome P450 isoforms involved in handling the antiretroviral agents. There have been cases reported of rhabdomyolysis and death presumably resulting from statin:PI interactions [6]. Because of the risk of drug-drug interaction in patients taking ART, a safer yet effective antihyperlipidemic therapy is desirable.

Dried oyster mushrooms (*Pleurotus ostreatus*), which are believed to contain a natural lovastatin-like compound, have been shown to provide significant cholesterol reductions in animal models. Administering a 5% dried *P. ostreatus *powder to male rats, decreased serum and liver cholesterol 33% and 27%, respectively [7]. Again in male rats, oyster mushrooms reduced HMG-CoA reductase by more than 30% [8]. The addition of 10% dried fruiting bodies of oyster mushrooms to a rabbit diet containing 1% cholesterol reduced serum cholesterol by 65%, lowered both the incidence of atherosclerotic plaques and plaque size, and prevented atherogenic changes in the aorta and coronary arteries [9]. In a small trial in 5 human subjects, 10-15 grams per day of a whole dried European strain of *Pleurotus ostreatus *mushrooms were ingested over 4 weeks, yielding a 30% reduction in LDL cholesterol levels [10]. The preclinical evidence and this small human trial, combined with the perceived need to find additional safe therapies for ART-associated hyperlipidemia, were the rationale for our pilot study.

## Methods

### Study Group

The study population was comprised of men and women 18 years of age or older with documented HIV infection referred from local physicians and newspaper advertisements. The study was approved by the Committee on Human Research at the University of California San Francisco. All participants signed the written informed consent form. The protocol was originally designed to include only patients who had been taking the protease inhibitor combination of lopinavir boosted by ritonavir (Kaletra); however, to facilitate accrual, the criteria were ultimately broadened to include those taking efavirenz (Sustiva) and/or any ritonavir-boosted protease inhibitor. Participants were required to have been taking the hyperlipidemia-inducing antiviral therapy for at least 12 weeks prior to study entry. Necessary laboratory values within 30 days prior to enrollment included serum non-HDL cholesterol levels of 160 mg/dL or higher and alanine and aspartate transaminase levels within 2.5 times the upper normal limit. Concurrent antihyperlipidemic treatment was not allowed, although prior treatment was not exclusionary. Pregnant and breastfeeding women were ineligible. Individuals with a documented history of diabetes mellitus, rhabdomyolysis, or statin-induced myopathy or myalgias were excluded.

### Study Medication

Fungi Perfecti, LLC (Olympia, Washington), produced a clone of a southern Californian strain of *Pleurotus ostreatus *collected from an oak log (*Quercus *sp.) that was grown in liquid culture for 3 days, inoculated into sterilized rice, incubated for two weeks and subsequently inoculated onto sterilized alder (*Alnus rubra*) sawdust. The young mushrooms were harvested, flash frozen, freeze-dried and "Fitz-milled" into a 20-80 mesh particle size. The resulting powder was divided into individual 15-gram packets, which subjects were instructed to add to soup or other hot food once daily for the 8-week study duration. The packets were maintained by the Investigational Pharmacy, Positive Health Program, University of California San Francisco, and distributed to subjects by the study coordinator or the nursing staff at the San Francisco General Hospital Clinical Research Center (CRC).

### Research Design and Procedures

This was a single-arm, open-label, 8-week proof-of-concept study of 20 subjects taking efavirenz or a ritonavir-boosted protease inhibitor based ART regimen who had elevated non-HDL cholesterol levels (160 mg/dL or higher). We chose the 8-week study duration because pharmacologic statins are known to exert an effect at 4 to 6 weeks, and we theorized that the lipid-lowering effect of the mushrooms would be similar or somewhat slower. The efficacy of *P. ostreatus *mushrooms in this population was investigated by measuring the effect size, variance, and time to maximum treatment effect.

Blood was drawn at screening, baseline, and every two weeks throughout the study to measure fasting lipids (total cholesterol, HDL cholesterol, direct LDL cholesterol, VLDL cholesterol, and triglycerides), liver enzymes, and creatine phosphokinase. A dietary history was obtained at Days 0, 28, and 56, by a Registered Dietician, to assess the specific composition of each subject's fat intake as closely as possible. Subjects were queried at Day 0 and each visit thereafter regarding adverse experiences, concomitant medications, and adherence to study medication. Safety measurements also included serial administration of a myopathy questionnaire to ascertain the development of any symptoms suggestive of muscle damage.

### Statistical Analysis

The primary objective was to test the hypothesis of no effect of oyster mushrooms on non-HDL-cholesterol. A subsequent randomized controlled trial would be worth pursuing if the true success rate, *ς*, estimated by *s/n*, the proportion of subjects who responded successfully in the pilot study, was 0.5 or more. For an individual patient, success was defined as a 30 mg/dL reduction in non-HDL cholesterol, comparing day 56 with day 0. With 20 patients, we had 80% power to reject H_0_: *ς *= 0.5 using a two-sided 0.05-level *z*-statistic (i.e., based on *z *= 1.96) if the true value was < 0.30. Exact 95% confidence intervals on *ς *according to the number of successes observed are reflected in Table 1. Therefore, we would not proceed with the RCT if we observed a sustained 30 mg/dL reduction in non-HDL cholesterol in 5 or fewer individuals.

**Table 1:** Success rate and 95% confidence Intervals based on number of patients out of 20 (*ς*) who achieve 30 mg/dL or greater reduction in non-HDL cholesterol

Number of successes, *s*:	5	6	7	8	9	10
**Estimated success rate, *s*/16:**	0.25	0.30	0.35	0.40	0.45	0.50
**95% CI on *ς*:**	(0.11, 0.47)	(0.15, 0.52)	(0.18, 0.57)	(0.22, 0.62)	(0.26, 0.66)	(0.30, 0.70)

During the intervention phase of the study, lipid levels were measured on days 0, 14, 28, 42, and 56. We used change from day 0 in non-HDL-cholesterol to test the null hypothesis of no lipid lowering effect of oyster mushrooms on non-HDL-cholesterol. The data were summarized as

where non-HDL_1 _represented the lipid level on day 0. We estimated mean within-person change in non-HDL-cholesterol at day 14, 28, 42 and 56 using generalized estimating equations to account for repeated measures on individuals. We described findings by plotting estimated mean (over subjects) lipid level (non-HDL_j_) and change in lipid level (C_j_) on each study day. We considered a 30 mg/dL decrease in non-HDL-cholesterol to be clinically significant. Hence, for each subject, we identified the first day on which a 30 mg/dL reduction in non-HDL-cholesterol was observed (*j *such that C_j _< -30 mg/dL), and obtained a Kaplan-Meier estimate of this time.

We also measured total cholesterol, HDL-cholesterol, direct LDL-cholesterol, and triglycerides. We used similar methods to estimate change in these estimates of lipid levels.

We report the proportion of patients with increases in muscle aches, liver enzymes, and CPK levels during the intervention phase of the study. We also report the occurrence of myopathy, transaminitis, or rhabdomyolysis in study patients. Adverse events were classified by stage based on the NIH Division of AIDS toxicity table for grading adverse events [11].

## Results

### Patient Characteristics

**Figure 1:**
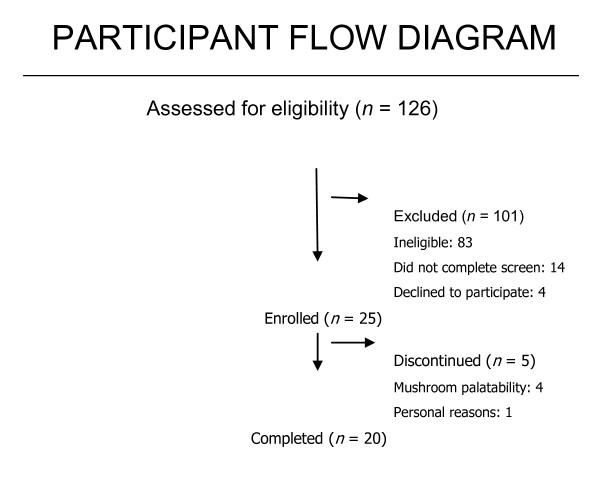
**Study Participant Flow Sheet**.

Of 126 individuals who were screened, 25 subjects were enrolled (Figure 1). Eighty-three patients failed to meet screening criteria; 14 did not complete the screen process; 4 who were eligible chose not to participate. Five enrolled subjects discontinued prior to study completion - 2 prior to day 14 and 3 between days 14 and 28- but were included in the intent-to-treat analysis. Participants were mostly male (96%) and Caucasian, non-Latino (68%), with a mean age of 46.4 years (Table 2). The mean baseline CD4+ T lymphocyte cell count was 492 cells/mm^3^, and on average subjects had been diagnosed with HIV disease for 13.7 years. Twenty patients were on boosted protease inhibitor based antiretroviral therapy regimens; 5 were on the non-nucleoside reverse transcriptase inhibitor, efavirenz, without an accompanying boosted protease inhibitor.

**Table 2:** Baseline Characteristics of Enrolled Participants (N = 25)

Participant Characteristic	n	Mean	%
**Years of age**		46.4	
**Male gender**	24		96
**Race/ethnicity**			
Caucasian	17		68
African American	4		16
Latino	4		16
**Years HIV-infected**		13.7	
**CD4 cell count**		494/mm^3^	
**HIV RNA < 75 copies/mL**	19		76
**Months on HAART**		82.3	
**Target dyslipidemia-inducing agents**			
lopinavir/ritonavir	15		60
atazanavir/ritonavir	3		12
tipranavir/ritonavir	1		4
fosamprenavir/ritonavir	1		4
efavirenz	5		20
**Months on target agent**		19.0	
**History of opportunistic infection**	13		52
**Cardiac risk factors**			
Lipodystrophy	13		52
hypertension	7		28
family history of hypertension	9		36
family history of hyperlipidemia	11		44
tobacco smokers	8		32
former smokers	7		28
alcohol users	15		60
former alcohol users	5		20
illicit substance users	14		56
former illicit substance users	7		28
**Prior antihyperlipidemic use:**	9		36

### Effect on Non-HDL Cholesterol, HDL cholesterol and triglycerides

At baseline, the mean non-HDL cholesterol was 204.5 mg/dL (normal range 100-199 mg/dL).

**Figure 2:**
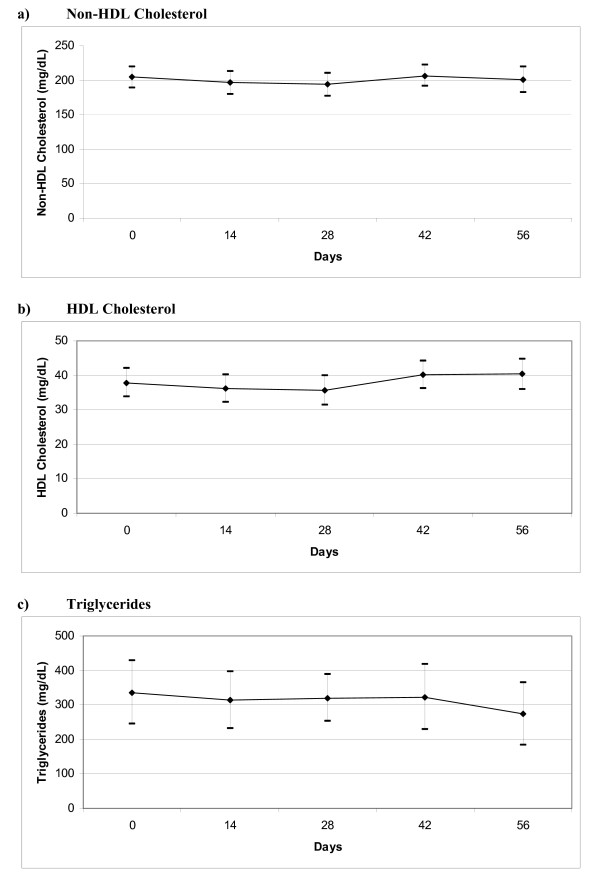
**Mean estimated lipid levels (with 95% confidence intervals) associated with use of oyster mushrooms**.

**Figure 3:**
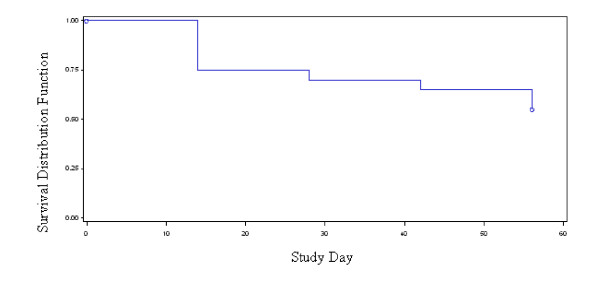
**Kaplan-Maier estimates of time to 30 mg/dL or greater reduction in non-HDL cholesterol**. Legend: ----- Product Limit Estimate Curve; o o o Censored Observations.

Figure 2a plots the serial values through day 56 of the study. The non-HDL cholesterol was essentially unchanged by the ingestion of the study preparation over the 8 weeks of the trial (Table 3). Nine of the participants achieved a clinically significant (30 mg/dL or greater) reduction in non-HDL cholesterol at some time during study follow-up (Figure 3). All of these patients were on a boosted PI ART regimen; none of the 5 NNRTI treated participants achieved a reduction of 30 mg/dL in non-HDL cholesterol. However, a clinically significant reduction was observed in only three patients at the end of study follow-up.

**Table 3:** Mean estimated within-person changes (with 95% confidence intervals) associated with use of oyster mushrooms*

	Day 14 mean change (95% CI)	Day 28 mean change (95% CI)	Day 42 mean change (95% CI)	Day 56 mean change (95% CI)
**Total Cholesterol**	-9.72 (-20.66, 1.23)	-12.99 (-26.89, 0.91)	4.33 (-11.39, 20.04)	-1.70 (-17.41, 14.02)
**Non-HDL**	-8.06 (-18.72, 2.60)	-10.89 (-23.51, 1.73)	2.06 (-13.24, 17.35)	-4.25 (-19.06, 10.56)
**VLDL**	2.80 (-4.47, 10.06)	3.47 (-4.08, 11.03)	5.86 (-3.45, 15.16)	-4.07 (-10.73, 2.59)
**LDL**	-8.73 (-20.41, 2.96)	-5.10 (-17.19, 6.99)	2.01 (-12.23, 16.25)	6.89 (-9.29, 23.07)
**HDL**	-1.65 (-3.54, 0.23)	-2.10 (-5.05, 0.85)	2.27 (-0.70, 5.24)	2.55 (-0.07, 5.17)
**Triglycerides**	-22.79 (-102.56, 56.98)	-16.64 (-86.78, 53.50)	-13.64 (-100.67, 73.40)	**-62.98** **(-120.92, -5.05)**
**Glucose**	0.23 (-5.68, 6.13)	1.90 (-1.77, 5.57)	4.59 (-1.51, 10.68)	-0.12 (-6.25, 6.01)
**AST**	-1.37 (-5.60, 2.86)	-4.62 (-9.65, 0.41)	-2.20 (-7.34, 2.95)	-2.09 (-7.43, 3.26)
**ALT**	-1.31 (-4.22, 1.62)	-3.33 (-6.85, 0.18)	1.56 (-3.29, 6.41)	0.36 (-4.44, 5.15)

* Statistically significant (p < 0.05) changes noted in bold.

HDL cholesterol was slightly depressed at baseline to 37.8 mg/dL (normal range 40-57 mg/dL). Figure 2b shows the 5.8% increase in HDL cholesterol that resulted after 8 weeks of *Pleurotus ostreatus *ingestion. Serum triglycerides fell 18.7% from an average 336.4 mg/dL (normal range less than 149 mg/dL) to 273.4 mg/dL by week 8 (Figure 2c). Models in which we forced a linear relationship over time did not yield statistically significant changes in any of our endpoints of interest (data not shown).

### Tolerability and Safety Considerations

Many participants described the mushroom preparation as difficult to dissolve and hence unpleasant to consume. They described a grittiness and earthy taste that may have contributed to decreased adherence to the daily study medication dosing. Other than the distaste, no significant adverse events were experienced. Safety laboratory measurements including serum glucose levels, hepatic transaminases and creatine phosphokinase to detect subclinical muscle damage remained stable throughout the eight weeks of the study (Table 3).

## Discussion

The particular preparation of freeze-dried *Pleurotus ostreatus *that was selected for evaluation in this pilot study failed to have a sustained effect on non-HDL cholesterol, the primary endpoint. Although 9 participants achieved the target 30 mg/dL reduction in non-HDL cholesterol at some time point in the study, only 3 had this reduction measured at study end. Therefore pursuit of a larger follow-on study was not deemed warranted based on our observed results. Of note, the study was initially designed to examine the effect in patients on a particular boosted protease inhibitor-based ART regimen that had been associated with hyperlipidemia. In order to assure that we would meet our target enrollment, the eligibility criteria was modified to allow participants on any boosted protease inhibitor as well as patients on the NNRTI efavirenz, which had also been shown to cause lipid perturbations, to be enrolled. Although the numbers are too small to make any definitive statement, we did note with interest that all of the non-HDL cholesterol declines of 30 mg/dL or greater occurred only in the participants on boosted PI regimens who overall experienced a reduction of non-HDL of 11.2 mg/dL compared to an increase of 13.3 mg/dL in the 5 efavirenz treated participants. This finding may be random or suggest the possibility that the intervention could work better with hyperlipidemia secondary to boosted PI regimens.

Trends suggesting an increase in HDL cholesterol and a statistically significant decrease in total triglycerides were noted but were not clinically meaningful. The mushroom preparation was clinically safe, without adverse effects other than being deemed distasteful by a number of study participants. This unpalatability may have led to decreased adherence to the daily ingestion of the study preparation which, in turn, may have influenced the results. Carbohydrate analysis by Stamets previously showed that the same strain of *Pleurotus ostreatus *mushrooms grown on sterilized sawdust had approximately 5% less sugars than the same strain grown on wheat straw [12]. This decrease in sugar content may have contributed to the unpalatability reported by some of the patients. Moreover, it is not known what effect the substrate and the corresponding changes in carbohydrate constitution would have on the outcome in reducing non-HDL cholesterol. Sterilized sawdust was chosen over pasteurized straw to assure a product of greater hygienity, since the colony forming units (cfu) of microbes on sterilized sawdust was < 10 cfu/gram whereas the surviving microbes on pasteurized straw was > 100,000 cfu/gram. The possible transference of microbes to the mushrooms was a concern given the study population of immunocompromised patients.

It is also possible that the species used and its preparation may have contributed to our negative results, although a similar dose of *Pleurotus ostreatus *was used in the earlier human trial that reported a 30% reduction in LDL-cholesterol. It may be, as noted above, that inoculating *Pleurotus *onto something other than sawdust could potentially increase the lipid-lowering potency of the preparation. Alternatively, the mushrooms being a natural product, the gentler lipid-lowering effect might not have had time to manifest during our 8-week study. This seems unlikely, however, in that the prior human study demonstrating a reduction was of four weeks' duration.

Other fungal products have already been demonstrated to have effectiveness in lowering lipid levels. Red yeast rice is the fermented byproduct of cooked rice in which a strain of *Monascus purpureus *is grown. Red yeast rice has been used in China for centuries as a medicinal food to promote "blood circulation" in the Traditional Chinese Medicine sense of the terms. Red yeast rice contains 9 monacolins, substances with HMG-Co-A reductase activity. Among the monacolins identified in red yeast rice is monacolin K, a naturally occurring form of mevinolin. Placebo controlled clinical trials have demonstrated a lipid-lowering effect of red yeast rice preparations in HIV negative patients [13,14]. The preparations are well-tolerated with mild headache and gastrointestinal distress as adverse effects with no evidence of impact on liver functions or muscle enzymes.

A recent trial has demonstrated the effectiveness of red yeast rice in hyperlipidemic patients intolerant of conventional statins [15]. To date, no clinical trials of red yeast rice in HIV patients on antiretroviral therapy have been reported. In view of the possibility of a monacolin interaction with antiviral agents through a cytochrome P450 mechanism, such a study with close safety monitoring may be warranted in the search for additional lipid-lowering agents compatible with HIV therapies.

## Conclusions

*Pleurotus ostreatus *as administered in this experiment did not lower non-HDL cholesterol in HIV patients with ART-induced hypercholesterolemia. Small changes in HDL and triglycerides were not of a clinical magnitude to warrant further study.

## Competing interests

Paul Stamets is the owner of Fungi Perfecti^® ^LLC. Fungi Perfecti^® ^LLC provided the custom-made, not commercially available mushroom product studied in this trial. There are no plans to make the product studied commercially available in the future. Fungi Perfecti^® ^LLC did not provide any funding for the trial. Other than Mr. Stamets', none of the contributing authors have any financial competing interests to report. There are no non-financial competing interests to declare in relation to this manuscript.

## Authors' contributions

DIA conceived of the study, obtained funding, oversaw the conduct of the trial and drafted the manuscript. PC screened, consented and enrolled the participants, coordinated specimen collection, study drug distribution and data collection and entry. SBS was involved in the conception of the study, oversaw data collection, conducted the data analyses and drafted the statistical section of the manuscript. MEK provided administrative and financial oversight for the study and the research team. NK-E was involved in study conception and authored the original application for funding to conduct the trial. PS was involved in the conception of the trial, produced and packaged the study medication and provided the study medication to the investigators to conduct the trial. All authors read, edited and approved the final manuscript.

## Pre-publication history

The pre-publication history for this paper can be accessed here:

http://www.biomedcentral.com/1472-6882/11/60/prepub
